# Risk Factors for Postoperative Sleep Disturbance in Elderly Patients Undergoing Hip Arthroplasty Surgery: The Role of Preoperative Depression and Anxiety

**DOI:** 10.62641/aep.v54i4.2244

**Published:** 2026-08-15

**Authors:** Qian Fang, Rong Li, Jinyan Xiao, Chenyu Liu

**Affiliations:** ^1^Department of Orthopaedics, Huanggang Central Hospital of Yangtze University, 438000 Huanggang, Hubei, China; ^2^Linshi Community Health Service Center, 408000 Chongqing, China; ^3^Medical Records Department, Huanggang Central Hospital of Yangtze University, 438000 Huanggang, Hubei, China; ^4^Department of Infectious Diseases, Huanggang Central Hospital of Yangtze University, 438000 Huanggang, Hubei, China

**Keywords:** hip arthroplasty, elderly, preoperative anxiety, preoperative depression, postoperative sleep disturbance, risk factors

## Abstract

**Background:**

Postoperative sleep disturbance (PSD) is a common complication following hip arthroplasty surgery (HAS), yet the role of preoperative psychological factors remains incompletely understood. This study aimed to identify risk factors for PSD in elderly patients undergoing HAS, with a specific focus on the impact of preoperative depression and anxiety.

**Methods:**

A case-control study was conducted on 204 elderly patients (age ≥ 60 years) who underwent HAS. Patients were categorized into PSD (n = 81) and non-PSD (n = 123) groups based on a Pittsburgh Sleep Quality Index (PSQI) global score > 7 at one-month follow-up. Data on demographics, preoperative psychological status (measured using the Self-Rating Anxiety Scale (SAS) and Self-Rating Depression Scale (SDS) as documented in medical records), perioperative clinical indicators, and postoperative sleep quality were extracted from electronic medical records. Univariate analyses, multivariable logistic regression, correlation analysis, and interaction assessments were performed.

**Results:**

Multivariable logistic regression identified four independent risk factors for PSD: higher preoperative SAS score (odds ratio (OR) = 1.14, 95% confidence interval (CI): 1.08–1.20, *p* < 0.001), higher preoperative SDS score (OR = 1.14, 95% CI: 1.08–1.21, *p* < 0.001), higher postoperative day 1 pain score (OR = 1.69, 95% CI: 1.13–2.53, *p* = 0.011), and longer time to first ambulation (OR = 1.13, 95% CI: 1.08–1.18, *p* < 0.001). Preoperative SAS and SDS scores were positively correlated with postoperative PSQI scores (r = 0.36 and r = 0.28, respectively, both *p* < 0.001). Stratified analysis confirmed preoperative anxiety/depression’s significant association with PSD regardless of preoperative sleep status. Preoperative anxiety and postoperative high pain showed a significant positive additive interaction (Relative Excess Risk due to Interaction (RERI) = 9.41, 95% CI: 2.77–34.12). The model had an area under curve (AUC) of 0.90 (95% CI: 0.85–0.94) and good calibration (Hosmer–Lemeshow *p* = 0.316).

**Conclusions:**

Preoperative depression and anxiety are significant independent risk factors for PSD in elderly HAS patients, exhibiting a synergistic effect with postoperative pain. These findings underscore the critical need to integrate routine preoperative psychological screening and targeted multidisciplinary interventions into perioperative care protocols to mitigate PSD and improve recovery outcomes.

## Introduction

As the global population ages, the incidence of conditions such as hip fractures and severe osteoarthritis has been rising year on year [1, 2]. Hip Arthroplasty surgery (HAS) has become the primary surgical intervention for improving joint function and alleviating pain in elderly patients [3]. Older patients, due to characteristics such as physiological decline and the presence of underlying conditions, experience a significantly higher incidence of postoperative complications compared to middle-aged and younger individuals [4]. Among these, postoperative sleep disturbance (PSD) is one of the most common problems, with reported incidence rates reaching 30% to 55% [5, 6]. PSD primarily manifest as difficulty falling asleep, maintaining sleep, and reduced sleep quality [7]. These issues not only prolong the duration of postoperative pain, delay wound healing and joint function recovery, but may also precipitate a series of secondary complications such as cognitive decline and impaired immune function [8]. Therefore, identifying risk factors for PSD in elderly patients undergoing HAS and developing targeted intervention strategies holds significant clinical importance for improving patient outcomes.

Currently, research into factors influencing PSD has predominantly centred on physiological and external factors such as surgical trauma stress, residual anaesthetic drugs, altered hospital environments, and postoperative pain [5]. However, the role of psychological factors, particularly the influence of negative emotional states prior to surgery, has not yet been fully elucidated or given due consideration. Although prior studies have linked preoperative psychological distress, postoperative pain, and postoperative sleep disturbance, few have specifically quantified their independent and synergistic effects in elderly hip arthroplasty patients, nor have they stratified risks by preoperative sleep status or validated a predictive model with high discriminative performance [9]. Elderly patients frequently experience negative emotions such as depression and anxiety prior to surgery, often stemming from concerns about surgical outcomes and uncertainties regarding post-operative recovery [10]. Anxiety and depression not only affect the preoperative physical condition of patients, but also may, through the neuro-endocrine-immune axis, intensify the postoperative stress response, thereby interfering with the sleep rhythm [11]. At present, for elderly patients undergoing HAS, the specific mechanism and impact intensity of preoperative depression and anxiety on PSD have not been fully clarified, and this study addresses these gaps by providing robust evidence for independent risk effects, synergistic interaction with pain, and consistent associations across preoperative sleep subgroups.

Based on the above background, this study adopted a retrospective analysis method, including the clinical data of elderly patients undergoing HAS. The focus was on exploring the correlation between preoperative depression, anxiety, and PSD. At the same time, other confounding factors were controlled to determine whether preoperative depression and anxiety were independent risk factors for PSD in elderly patients undergoing HAS. The findings of this study aim to supplement the clinical evidence regarding risk factors for postoperative sleep disorders in elderly hip replacement patients. They provide a theoretical basis for preoperative screening of high-risk individuals, implementing targeted psychological interventions and sleep management, thereby optimising perioperative clinical management protocols. Ultimately, this approach seeks to enhance patients’ postoperative sleep quality and rehabilitation outcomes.

## Materials and Methods

### Patient Population

This was a single-center retrospective study conducted at Huanggang Central Hospital of Yangtze University . We retrospectively reviewed data from elderly patients who underwent HAS (including total hip arthroplasty and hemiarthroplasty) between June 2022 and June 2025. All study data were extracted from electronic medical records, paper medical charts, and nursing records of the patients, including admission assessment forms, medical records, laboratory test reports, nursing evaluation records, and discharge summaries. Initially, 248 elderly patients who underwent hip arthroplasty were screened for eligibility. Among them, 19 patients were excluded due to incomplete perioperative data, 12 were excluded due to missing postoperative 1-month follow-up data, 8 were excluded due to violation of exclusion criteria, and 5 were excluded for other reasons. Finally, 204 patients were included in the final analysis, with a follow-up rate of 82.26% (204/248). No significant differences in baseline characteristics were observed between patients with complete follow-up and those lost to follow-up, suggesting that loss to follow-up had no substantial impact on the main study results.

### Inclusion and Exclusion Criteria

Inclusion criteria: (1) Age ≥ 60 years; (2) Underwent primary unilateral or bilateral hip arthroplasty due to femoral neck fracture, severe hip osteoarthritis, or avascular necrosis of the femoral head; (3) Successful completion of surgery with a postoperative hospital stay ≥ 3 days; (4) Complete medical and nursing records, allowing extraction of preoperative psychological status, preoperative sleep status, postoperative sleep status, and relevant baseline data. Exclusion criteria: (1) Preoperative severe cognitive impairment as determined by qualified geriatricians or neuropsychologists based on comprehensive clinical evaluation and the Mini-Mental State Examination (MMSE) [12], assessed within 48 hours prior to surgery, history of mental illness, or current use of antipsychotic drugs or regular use of sedative-hypnotic drugs within 2 weeks before surgery; (2) Comorbidity with end-stage diseases such as severe liver and kidney failure, heart failure, or malignant tumors; (3) Postoperative severe complications (e.g., infection, prosthesis dislocation, pulmonary embolism) that affected sleep assessment.

### Data Collection

Demographic and clinical data were retrospectively and independently extracted from the hospital’s electronic health record system by two trained researchers, and any discrepancies were resolved by a third senior investigator to ensure data accuracy and consistency. Data extraction encompassed preoperative, intraoperative, and postoperative variables. Preoperative data included age, gender, body mass index (BMI), educational level, Charlson Comorbidity Index (CCI), which was calculated according to the standard scoring criteria including 19 major comorbidities with weighted scores, as validated and widely used in perioperative elderly populations [13], diagnosis, and preoperative sleep status. Preoperative psychological status was measured using the Self-Rating Anxiety Scale (SAS) and the Self-Rating Depression Scale (SDS) as recorded in the medical records. Perioperative data included surgical type, anesthesia method, operation duration, intraoperative blood loss, postoperative pain scores (assessed by the Numerical Rating Scale (NRS)) on days 1, 2, and 3, time to first ambulation, Harris Hip Score (HHS) at discharge, and length of postoperative hospital stay. Hip function at discharge was evaluated using the HHS [14], a scale from 0 to 100, with higher scores indicating better function. Postoperative sleep quality was evaluated one month after surgery during the follow-up visit. Furthermore, information on patients’ preoperative anxiety, depression, and sleep status, collected within three days before surgery, was also recorded.

### Measurement Instruments

(1) Preoperative and postoperative sleep quality assessment: The Pittsburgh Sleep Quality Index (PSQI) was used [15]. This scale includes 7 dimensions: subjective sleep quality, sleep latency, sleep duration, sleep efficiency, sleep disturbances, use of sleep medication, and daytime dysfunction. Each dimension is scored from 0 to 3 points, with a total score ranging from 0 to 21 points. A total score > 7 points indicates sleep disturbance, and a higher score indicates worse sleep quality. This cutoff (> 7) was selected based on widely accepted diagnostic criteria for sleep disturbance in perioperative and elderly populations, as validated in previous hip arthroplasty studies [5]. In this study, the Cronbach’s α coefficient of the PSQI was 0.81. The scale had been completed 3 days before surgery to document preoperative sleep status and during the 1-month postoperative follow-up to document PSD, as extracted from the medical charts. Postoperative use of sleep-promoting medications was not restricted by study protocol and was administered at the discretion of the attending anesthesiologist and geriatrician according to routine clinical practice. No standardised sedative or hypnotic protocol was applied. The use of sleep medication was recorded as one component of the PSQI score and was not adjusted for as a separate covariate in the analysis. It should be noted that the diagnosis of PSD in this study was based solely on the PSQI global score cutoff (> 7), as a formal psychiatric evaluation according to standardized diagnostic systems (e.g., Diagnostic and Statistical Manual of Mental Disorders, Fifth Edition (DSM-5) criteria) was not available due to the retrospective nature of the study.

(2) Preoperative anxiety assessment: The SAS was used, which consists of 20 items, each scored from 1 to 4 points [16]. The standard score is calculated by multiplying the raw score by 1.25 and rounding to the nearest integer, ranging from 25 to 100 points. A standard score ≥ 50 points indicates the presence of anxiety symptoms [17]. In this study, the Cronbach’s α coefficient of the SAS was 0.85. The scale was administered 3 days before surgery.

(3) Preoperative depression assessment: The SDS was used, which includes 20 items, each scored from 1 to 4 points [18]. The standard score is obtained by multiplying the raw score by 1.25 and rounding to the nearest integer, ranging from 25 to 100 points. A standard score ≥ 53 points indicates the presence of depression symptoms [17]. In this study, the Cronbach’s α coefficient of the SDS was 0.80. The scale was administered 3 days before surgery.

(4) Postoperative pain assessment: The NRS was used, with scores ranging from 0 to 10 points (0 points = no pain, 10 points = most severe pain) [19]. Pain scores were recorded on postoperative days 1, 2, and 3 in the resting state. For interaction analysis, NRS scores on postoperative day 1 were dichotomized into low pain (≤ 3 points) and high pain (> 3 points).

### Statistical Analysis

All statistical analyses were performed using IBM SPSS Statistics (Version 26.0, IBM Corp., Armonk, NY, USA). Continuous variables were tested for normality first. Normally distributed data were expressed as mean ± standard deviation (mean ± SD), and between-group comparisons were performed using the independent samples *t*-test. Non-normally distributed data were expressed as median (interquartile range) [M (Q_1_, Q_3_)], and between-group comparisons were conducted using the Wilcoxon rank-sum test. Categorical variables were expressed as frequency (percentage) [n (%)], and between-group comparisons were performed using the χ^2^ test or Fisher’s exact test (when the theoretical frequency < 5). Univariate analysis was used to screen potential risk factors for PSD, and variables with *p* < 0.1 in univariate analysis were included in the multivariable Logistic regression model. Multicollinearity among candidate variables was evaluated using the variance inflation factor (VIF), with VIF < 5 indicating no significant multicollinearity. The backward stepwise regression method was adopted, with the removal criterion set at *p* > 0.10 based on the likelihood ratio test. Results were expressed as odds ratios (ORs) with 95% confidence intervals (CIs). For correlation analysis between preoperative psychological scores and postoperative PSQI scores, normality testing confirmed that the PSQI total score and its dimensions followed a normal distribution (Shapiro–Wilk test, *p* > 0.05), thus Pearson correlation analysis was used. The discriminative ability of the final logistic regression model was evaluated by calculating the area under the receiver operating characteristic (ROC) curve (AUC). Calibration was assessed using the Hosmer–Lemeshow goodness-of-fit test. For interaction analysis, multiplicative interaction was analyzed using Logistic regression in SPSS, with the product term of mood disorders (anxiety/depression) and postoperative pain (dichotomized) included in the model. Additive interaction was evaluated using the Excel calculation table developed by Andersson *et al*. [20] to calculate indicators including Relative Excess Risk due to Interaction (RERI), Attributable Proportion due to Interaction (AP), and Synergy Index (S). An additive interaction was considered significant if the 95% confidence interval (CI) of RERI did not include 0, and a multiplicative interaction was considered significant if the *p* value of the product term was < 0.05. A two-tailed *p* < 0.05 was considered statistically significant.

## Results

### Baseline Characteristics of Study Participants

A total of 204 elderly patients undergoing HAS were included in this case-control study. Of these, 81 patients were assigned to the PSD group, while 123 were assigned to the non-PSD group. The baseline demographic and clinical characteristics of the study population are presented in Table 1. No significant differences were observed between the PSD and non-PSD groups in terms of age, BMI, gender, educational level, Charlson Comorbidity Index, diagnosis, surgical type, or anesthesia method (*p* > 0.05).

**Table 1.** **Characteristics of study participants**.

Variables		Total	Non-PSD	PSD	Statistic	*p*
	(n = 204)	(n = 123)	(n = 81)			
Age, years, mean ± SD		74.92 ± 6.89	74.50 ± 6.97	75.56 ± 6.77	*t* = –1.07	0.284
BMI, kg/m^2^, mean ± SD		24.57 ± 3.26	24.42 ± 3.41	24.80 ± 3.04	*t* = –0.83	0.409
Gender, n (%)					χ^2^ = 0.18	0.674
	Male	77 (37.75)	45 (36.59)	32 (39.51)		
	Female	127 (62.25)	78 (63.41)	49 (60.49)		
Educational Level, n (%)					χ^2^ = 3.09	0.079
	High school or above	78 (38.24)	53 (43.09)	25 (30.86)		
	Below high school	126 (61.76)	70 (56.91)	56 (69.14)		
Charlson Comorbidity Index, M (Q_1_, Q_3_)		1.00 (0.00, 2.00)	1.00 (0.00, 2.00)	1.00 (1.00, 2.00)	*z* = –1.72	0.086
Diagnosis, n (%)					χ^2^ = 1.15	0.283
	Femoral neck fracture	132 (64.71)	76 (61.79)	56 (69.14)		
	Hip osteoarthritis	72 (35.29)	47 (38.21)	25 (30.86)		
Surgical Type, n (%)					χ^2^ = 1.11	0.293
	Hemiarthroplasty	64 (31.37)	42 (34.15)	22 (27.16)		
	Total hip arthroplasty	140 (68.63)	81 (65.85)	59 (72.84)		
Anesthesia Type, n (%)					χ^2^ = 0.19	0.660
	Neuraxial anesthesia	92 (45.10)	57 (46.34)	35 (43.21)		
	General anesthesia	112 (54.90)	66 (53.66)	46 (56.79)		

Abbreviations: PSD, postoperative sleep disturbance; BMI, body mass index; SD, standard deviation.

### Sleep Quality Assessment by PSQI

Table 2 presents the sleep quality scores of the two groups assessed by the PSQI. The median of total PSQI score in the PSD group was 10.00 (8.00, 12.00), which was significantly higher than that in the non-PSD group [6.00 (5.00, 7.00), *p* < 0.001]. Compared with the non-PSD group, the PSD group exhibited significantly higher scores in all PSQI dimensions, including subjective sleep quality (*p* < 0.001), sleep latency (*p* = 0.048), sleep duration (*p* < 0.001), sleep efficiency (*p* < 0.001), sleep disturbances (*p* = 0.005), use of sleep medication (*p* < 0.001), and daytime dysfunction (*p* < 0.001).

**Table 2.** **Sleep quality scores assessed by PSQI at one-month postoperative follow-up**.

Variables	Total	Non-PSD	PSD	Statistic	*p*
	(n = 204)	(n = 123)	(n = 81)		
Total score, M (Q_1_, Q_3_)	7.00 (6.00, 9.00)	6.00 (5.00, 7.00)	10.00 (8.00, 12.00)	*z* = –11.87	< 0.001
Subjective sleep quality, M (Q_1_, Q_3_)	1.00 (1.00, 2.00)	1.00 (0.00, 1.00)	2.00 (1.00, 2.00)	*z* = –5.12	< 0.001
Sleep latency, M (Q_1_, Q_3_)	1.00 (1.00, 2.00)	1.00 (0.50, 2.00)	1.00 (1.00, 2.00)	*z* = –1.98	0.048
Sleep duration, M (Q_1_, Q_3_)	1.00 (0.00, 2.00)	1.00 (0.00, 1.00)	2.00 (1.00, 3.00)	*z* = –6.10	< 0.001
Sleep efficiency, M (Q_1_, Q_3_)	1.00 (0.00, 2.00)	0.00 (0.00, 1.00)	2.00 (1.00, 2.00)	*z* = –6.10	< 0.001
Sleep disturbances, M (Q_1_, Q_3_)	1.00 (0.00, 2.00)	1.00 (0.00, 2.00)	1.00 (1.00, 2.00)	*z* = –2.84	0.005
Use of sleep medication, M (Q_1_, Q_3_)	0.00 (0.00, 1.00)	0.00 (0.00, 0.00)	1.00 (0.00, 2.00)	*z* = –6.29	< 0.001
Daytime dysfunction, M (Q_1_, Q_3_)	1.00 (0.00, 2.00)	1.00 (0.00, 2.00)	1.00 (1.00, 2.00)	*z* = –3.58	< 0.001

Abbreviations: PSD, postoperative sleep disturbance; PSQI, Pittsburgh Sleep Quality Index; M, median; Q₁, first quartile; Q₃, third quartile.

### Preoperative Psychological Status and Its Correlation With Postoperative Sleep Quality

**Fig. 1.**
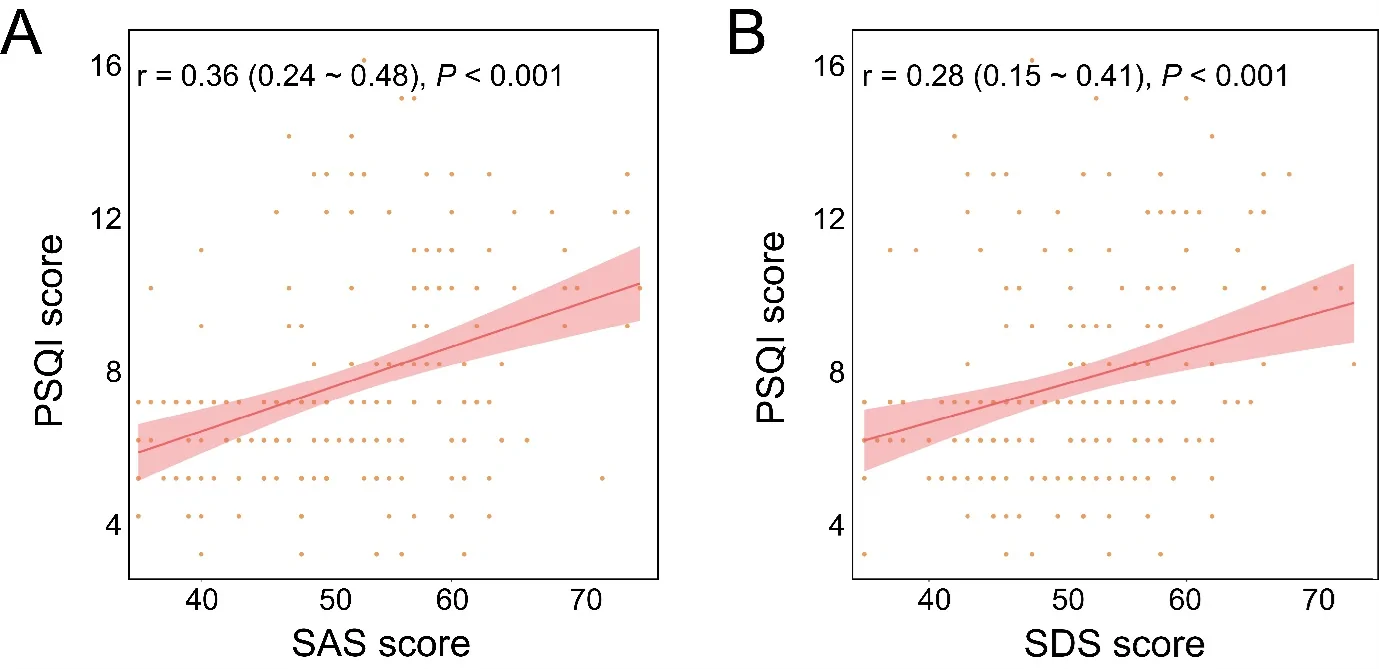
**Correlation between preoperative psychological scores and postoperative PSQI scores**. (A) SAS and PSQI. (B) SDS and PSQI. SAS, Self-Rating Anxiety Scale; SDS, Self-Rating Depression Scale; PSQI, Pittsburgh Sleep Quality Index.

Significant differences in preoperative psychological status were found between the two groups (Table 3). The mean SAS score in the PSD group (56.80 ± 8.28) was significantly higher than that in the non-PSD group (48.41 ± 7.88, *p* < 0.001), as was the mean SDS score (55.11 ± 7.95 vs. 48.67 ± 7.28, *p* < 0.001). The proportions of patients with anxiety (81.48% vs. 41.46%, *p* < 0.001) and depression (64.20% vs. 32.52%, *p* < 0.001) symptoms were also significantly higher in the PSD group. Correlation analysis revealed significant positive correlations between preoperative psychological scores and postoperative PSQI scores (Fig. 1). Preoperative SAS scores were positively correlated with postoperative PSQI scores (r = 0.36, 95% CI: 0.24–0.48, *p* < 0.001) (Fig. 1A), and preoperative SDS scores also showed a positive correlation with postoperative PSQI scores (r = 0.28, 95% CI: 0.15–0.41, *p* < 0.001) (Fig. 1B), indicating that higher preoperative anxiety and depression levels are associated with worse postoperative sleep quality.

**Table 3.** **Comparison of psychological status**.

Variables		Total	Non-PSD	PSD	Statistic	*p*
	(n = 204)	(n = 123)	(n = 81)			
SAS score, mean ± SD		51.74 ± 9.02	48.41 ± 7.88	56.80 ± 8.28	*t* = –7.30	< 0.001
SDS score, mean ± SD		51.23 ± 8.17	48.67 ± 7.28	55.11 ± 7.95	*t* = –5.95	< 0.001
Anxiety symptom, n (%)					χ^2^ = 31.98	< 0.001
	No	87 (42.65)	72 (58.54)	15 (18.52)		
	Yes	117 (57.35)	51 (41.46)	66 (81.48)		
Depression symptom, n (%)					χ^2^ =19.79	< 0.001
	No	112 (54.90)	83 (67.48)	29 (35.80)		
	Yes	92 (45.10)	40 (32.52)	52 (64.20)		

Abbreviations: PSD, postoperative sleep disturbance; SAS, Self-Rating Anxiety Scale; SDS, Self-Rating Depression Scale; SD, standard deviation.

### Comparison of Perioperative Indicators

As shown in Table 4, no significant differences were observed between the two groups in operation duration (*p* = 0.385), intraoperative blood loss (*p* = 0.522), postoperative day 2 pain score (*p* = 0.925), postoperative day 3 pain score (*p* = 0.421), or postoperative hospital stay (*p* = 0.113). However, the PSD group had a significantly higher postoperative day 1 pain score (4.56 ± 1.11 vs. 3.97 ± 0.95, *p* < 0.001), longer time to first ambulation (46.11 ± 10.49 vs. 37.87 ± 7.42 hours, *p* < 0.001), and lower Harris Hip Score at discharge (67.35 ± 6.34 vs. 71.38 ± 7.01, *p* < 0.001) compared to the non-PSD group.

**Table 4.** **Comparison of perioperative indicators**.

Variables	Total	Non-PSD	PSD	Statistic	*p*
	(n = 204)	(n = 123)	(n = 81)		
Operation duration, minutes, mean ± SD	95.65 ± 19.18	96.60 ± 19.36	94.21 ± 18.92	*t* = 0.87	0.385
Intraoperative blood loss, ml, mean ± SD	353.19 ± 126.80	348.56 ± 129.48	360.21 ± 123.08	*t* = –0.64	0.522
Postoperative day 1 pain score, mean ± SD	4.20 ± 1.05	3.97 ± 0.95	4.56 ± 1.11	*t* = –4.05	< 0.001
Postoperative day 2 pain score, mean ± SD	4.09 ± 1.28	4.08 ± 1.30	4.10 ± 1.26	*t* = –0.09	0.925
Postoperative day 3 pain score, mean ± SD	3.49 ± 1.12	3.44 ± 1.12	3.57 ± 1.11	*t* = –0.81	0.421
Time to first ambulation, hours, mean ± SD	41.14 ± 9.63	37.87 ± 7.42	46.11 ± 10.49	*t* = –6.13	< 0.001
Harris Hip Score at discharge, mean ± SD	69.78 ± 7.02	71.38 ± 7.01	67.35 ± 6.34	*t* = 4.18	< 0.001
Postoperative hospital stay, days, mean ± SD	6.44 ± 1.32	6.32 ± 1.26	6.62 ± 1.41	*t* = –1.59	0.113

Abbreviations: PSD, postoperative sleep disturbance; SD, standard deviation.

### Independent Risk Factors for PSD

The variables with *p* < 0.1 from the single-factor analysis were included in the multivariate Logistic regression model, and the backward stepwise method was adopted. All candidate variables had VIF values < 5, indicating no significant multicollinearity. Table 5 presents the results of multivariable logistic regression analysis. The results show that preoperative SAS score (odds ratio (OR) = 1.14, 95% CI: 1.08–1.20, *p* < 0.001), preoperative SDS score (OR = 1.14, 95% CI: 1.08–1.21, *p* < 0.001), postoperative day 1 pain score (OR = 1.69, 95% CI: 1.13–2.53, *p* = 0.011), and time to first ambulation (OR = 1.13, 95% CI: 1.08–1.18, *p* < 0.001) were identified as independent risk factors for PSD.

**Table 5.** **Independent risk factors for PSD identified by multivariable logistic regression**.

Variables	β	SE	*z*	*p*	OR (95% CI)
Intercept	–21.34	2.93	–7.29	< 0.001	
SAS score	0.13	0.03	4.84	< 0.001	1.14 (1.08–1.20)
SDS score	0.13	0.03	4.63	< 0.001	1.14 (1.08–1.21)
Postoperative day 1 pain score	0.52	0.21	2.53	0.011	1.69 (1.13–2.53)
Time to first ambulation	0.12	0.02	5.08	< 0.001	1.13 (1.08–1.18)

Abbreviations: PSD, postoperative sleep disturbance; SE, standard error; OR, odds ratio; CI, confidence interval; SAS, Self-Rating Anxiety Scale; SDS, Self-Rating Depression Scale.

### Evaluation of the Multivariate Regression Model.

**Fig. 2.**
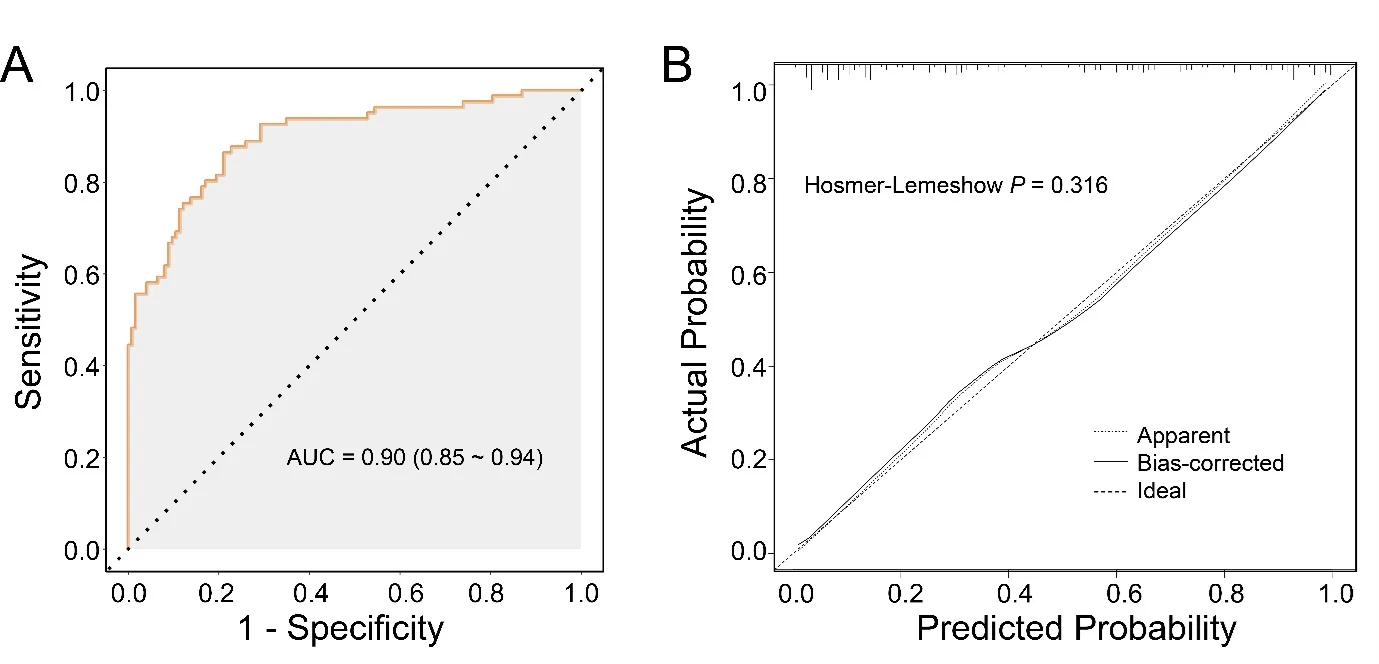
**Performance of the predictive model for PSD**. (A) ROC curve. (B) Calibration curve. PSD, postoperative sleep disturbance; ROC, receiver operating characteristic.

The final multivariable model demonstrated excellent discriminative ability for predicting PSD. The AUC under the ROC curve was 0.90 (95% CI: 0.85–0.94) (Fig. 2A). Calibration of the model, assessed by the Hosmer–Lemeshow goodness-of-fit test, indicated no significant deviation between predicted and observed probabilities (χ^2^ = 8.56, *p* = 0.316), as visualized in the calibration plot (Fig. 2B).

### Stratified Analysis by Preoperative Sleep Status

Stratified analysis based on preoperative sleep status (Table 6) showed that among patients with preoperative sleep disturbances (n = 61), preoperative SAS score (OR = 1.20, 95% CI: 1.06–1.35, *p* = 0.003), preoperative SDS score (OR = 1.12, 95% CI: 1.01–1.25, *p* = 0.042), and time to first ambulation (OR = 1.19, 95% CI: 1.05–1.33, *p* = 0.005) were significantly associated with PSD, while postoperative day 1 pain score was not (OR = 1.59, 95% CI: 0.68–3.73, *p* = 0.287). Among patients without preoperative sleep disturbances (n = 143), all four factors—preoperative SAS score (OR = 1.11, 95% CI: 1.05–1.18, *p* < 0.001), preoperative SDS score (OR = 1.15, 95% CI: 1.08–1.24, *p* < 0.001), postoperative day 1 pain score (OR = 1.67, 95% CI: 1.04–2.67, *p* = 0.033), and time to first ambulation (OR = 1.11, 95% CI: 1.05–1.17, *p* < 0.001)—were significantly associated with PSD.

**Table 6.** **Stratified analysis of risk factors for PSD by preoperative sleep status**.

Variables	β	SE	*z*	*p*	OR (95% CI)
Patients with preoperative					
sleep disturbances (n = 61)					
SAS score	0.18	0.06	2.95	0.003	1.20 (1.06–1.35)
SDS score	0.11	0.06	2.03	0.042	1.12 (1.01–1.25)
Postoperative day 1 pain score	0.46	0.44	1.06	0.287	1.59 (0.68–3.73)
Time to first ambulation	0.17	0.06	2.83	0.005	1.19 (1.05–1.33)
Patients without preoperative					
sleep disturbances (n = 143)					
SAS score	0.11	0.03	3.64	< 0.001	1.11 (1.05–1.18)
SDS score	0.14	0.04	4.04	< 0.001	1.15 (1.08–1.24)
Postoperative day 1 pain score	0.51	0.24	2.13	0.033	1.67 (1.04–2.67)
Time to first ambulation	0.10	0.03	3.97	< 0.001	1.11 (1.05–1.17)

Abbreviations: PSD, postoperative sleep disturbance; SE, standard error; OR, odds ratio; CI, confidence interval; SAS, Self-Rating Anxiety Scale; SDS, Self-Rating Depression Scale.

### Interaction Effects Between Mood Disorders and Postoperative Pain

Table 7 shows no significant multiplicative interaction between anxiety and postoperative day 1 pain (OR = 2.49, 95% CI: 0.60–10.34, *p* = 0.210) or between depression and postoperative day 1 pain (OR = 1.13, 95% CI: 0.32–4.03, *p* = 0.848). For additive interaction (Table 8), anxiety and postoperative pain showed a significant positive additive interaction (RERI = 9.41, 95% CI: 2.77–34.12; AP = 0.68, 95% CI: 0.30–0.88; S = 3.79, 95% CI: 1.47–16.53). For depression and postoperative pain, a potential positive additive interaction was observed but did not reach statistical significance (RERI = 8.32, 95% CI: –0.63–40.19; AP = 0.57, 95% CI: –0.07–0.86; S = 2.60, 95% CI: 0.91–10.24).

**Table 7.** **Multiplicative interaction effects between mood disorders and postoperative pain on PSD**.

Independent Variable	Anxiety		Depression	
	OR (95% CI)	*p*	OR (95% CI)	*p*
Mood disorder	3.98 (1.72–9.21)	0.001	3.90 (1.73–8.80)	0.001
Pain^a^	1.40 (0.42–4.61)	0.585	3.29 (1.37–7.89)	0.008
Mood disorder × Pain	2.49 (0.60–10.34)	0.210	1.13 (0.32–4.03)	0.848

^a^Postoperative day 1 pain score > 3. Abbreviations: PSD, postoperative sleep disturbance; OR, odds ratio; CI, confidence interval.

**Table 8.** **Additive interaction effects between mood disorders and postoperative pain on PSD**.

Mood	Pain level	Anxiety		Depression	
disorder		OR (95% CI)	*p*	OR (95% CI)	*p*
Yes	> 3	13.78 (5.60–33.92)	< 0.001	14.50 (5.46–38.54)	< 0.001
Yes	≤ 3	3.97 (1.72–9.21)	0.001	3.90 (1.73–8.80)	0.001
No	> 3	1.39 (0.42–4.61)	0.585	3.29 (1.37–7.89)	0.008
No	≤ 3	Ref	Ref	Ref	Ref
RERI		9.41 (2.77–34.12)		8.32 (–0.63–40.19)	
AP		0.68 (0.30–0.88)		0.57 (–0.07–0.86)	
S		3.79 (1.47–16.53)		2.60 (0.91–10.24)	

Abbreviations: PSD, postoperative sleep disturbance; OR, odds ratio; CI, confidence interval; Ref, reference group; RERI, relative excess risk due to interaction; AP, attributable proportion due to interaction; S, synergy index.

## Discussion

This study systematically investigated the independent association of preoperative depression and anxiety on PSD through a retrospective analysis of 204 elderly patients undergoing HAS. The study found that the incidence of PSD within one month after surgery was 39.7%. Multivariate Logistic regression analysis ultimately identified four independent risk factors: preoperative anxiety score, preoperative depression score, pain score on the first postoperative day, and the time of the first postoperative ambulation. This indicates that, in addition to the known physiological stress (such as early pain, delayed activity), the patient’s preoperative psychological state is a key and independent dimension for predicting postoperative sleep quality. Correlation analysis further supported that preoperative anxiety, depression, and postoperative PSQI score were significantly positively correlated. Stratified analysis showed that preoperative anxiety and depression were significantly associated with PSD regardless of whether the patient had sleep disorders before the surgery. More in-depth interaction analysis revealed a significant additive interaction between anxiety and high postoperative pain levels, meaning that when both exist simultaneously, their risk for PSD has a synergistic amplification effect. These results collectively constructed a multi-dimensional risk map that includes psychological, physiological, and rehabilitation behaviors, emphasizing the necessity of integrating psychological assessment and intervention in perioperative management.

The core finding of this study was that preoperative anxiety and depression were independently associated with an increased risk of PSD. Traditionally, postoperative sleep disorders have been attributed to direct physiological stimuli such as hospital environment, residual anesthetic drugs, surgical trauma and pain [21, 22, 23]. In a cross-sectional study involving 759 patients, Liu and Tang [5] reported that early postoperative pain, hospital environment, and the use of dexmedetomidine were significantly associated with PSD. The research conducted by Driesman *et al*. [24] indicates that the use of opioid drugs by patients after hip replacement surgery is associated with poorer sleep quality. In this study, the time point for extracting sleep data was when the patients returned to the hospital for a follow-up visit one month after the surgery. This time point effectively avoided confounding factors such as anesthetic drugs and surgical stress. Our data show that even after controlling for confounding factors such as pain and mobility, for every additional point increase in the SAS and SDS scores, the risk of PSD still increases by 14%. There have been similar reports in the past. For instance, Wang *et al*. [25] found that anxiety was a risk factor for PSD in patients undergoing total hip replacement surgery. From a pathophysiological perspective, preoperative anxiety and depression may be potentially linked to neuroendocrine-immune axis regulation, including activation of the hypothalamic-pituitary-adrenal axis and increased cortisol secretion, which may in turn be associated with disturbed sleep-wake rhythms [26, 27]. Furthermore, previous studies have shown that anxiety and depression are associated with higher postoperative pain scores [11, 28]. Pain, as another independent risk factor in this study, synergises with psychological factors to further exacerbate sleep disturbances. Correlation analysis revealed that SAS scores showed a stronger association with PSQI scores than SDS scores, suggesting that preoperative anxiety may exert a more pronounced effect on postoperative sleep. This finding may be closely related to the unique physiological and psychological characteristics of elderly patients. Compared with depression, preoperative anxiety often manifests as acute apprehension, fear of surgery, and worry about postoperative dysfunction, which can rapidly activate the sympathetic nervous system, increase catecholamine levels, elevate heart rate and alertness, and directly disrupt sleep initiation and maintenance [29]. In elderly individuals, autonomic regulatory function is naturally diminished, making them more vulnerable to anxiety-induced sleep disturbances. In contrast, depression is characterized by sustained low mood and apathy, which tend to exert a milder, more chronic influence on sleep rather than acute disruption [27]. These differences may explain the stronger correlation between anxiety and PSD observed in this study. Extended time to first ambulation also constitutes an independent risk factor for pressure ulcers, a finding that adds to the evidence linking perioperative functional recovery with sleep quality. Early postoperative ambulation not only promotes the recovery of limb functions, but also helps regulate the biological rhythm, reduce pain perception and improve sleep. However, for PSD patients, poor sleep quality may further delay their ambulation, thus creating a vicious cycle [30]. Notably, this study assessed PSD at 1 month postoperatively, whereas acute pain, anesthesia, and environmental factors mainly influence early sleep within days to one week after surgery. This timing difference indicates that the observed sleep disturbance at one month represents persistent or chronic sleep impairment, rather than only acute surgical effects. Preoperative psychological distress may act as a stable vulnerability factor leading to prolonged sleep disruption, which underscores the clinical importance of long‑term sleep monitoring after hip arthroplasty [7].

Another important finding is that, after stratifying by preoperative sleep status, the associations between preoperative anxiety, depression and PSD remained significant. This fully confirms the robustness and independence of preoperative psychological factors in influencing postoperative sleep. Among patients with preoperative sleep disorders, the preoperative SAS score and SDS score remained independent risk factors for PSD, while postoperative pain did not have a significant impact. A possible explanation is that patients with preoperative sleep disorders represent a high-risk subgroup with compromised baseline sleep health. In such patients, preoperative psychological distress may serve as a more dominant and persistent determinant of postoperative sleep outcomes compared with acute postoperative pain, which is consistent with reports that preoperative mental health and baseline sleep status carry greater weight for long-term postoperative sleep and recovery than transient acute pain stimuli [31, 32]. On the contrary, in patients without preoperative sleep disorders, preoperative anxiety, depression, and postoperative pain all significantly affected PSD, indicating that for patients with intact sleep function, psychological stress and physical pain can both be triggering factors for sleep disorders. This stratification result is consistent with the conclusion of O’Connor *et al*.’s systematic review [10], which states that preoperative psychological factors have no limit on the impact on postoperative outcomes based on the baseline health status. At the same time, it further refined the risk differences under different baseline sleep states, providing a basis for individualized intervention. Furthermore, the OR values of preoperative psychological factors in both groups were greater than 1, and the confidence intervals did not include 1. This indicates that regardless of the patients’ preoperative sleep conditions, intervening to reduce preoperative anxiety and depression holds significant clinical value [33]. Another finding that deserves in-depth discussion is the significant additive interaction between anxiety and postoperative pain. In patients undergoing orthopedic surgeries, preoperative anxiety is widespread and has been found to be associated with poorer postoperative outcomes [34]. Çalışkan and Aksoy [35] observed a significant association between preoperative anxiety and postoperative pain levels in the population undergoing hip replacement surgery. From a pathophysiological perspective, anxiety may be associated with increased pain perception through a central sensitization mechanism, reducing the tolerance threshold for postoperative traumatic pain in patients [36, 37]. And pain, as a powerful physiological stressor, will further activate the sympathetic nervous system, which may contribute to a vicious cycle of “anxiety–pain–sleep disorder” [38]. RERI = 9.41 and the 95% CI does not include 0, suggesting that this synergistic effect is statistically significant. Meanwhile, AP = 0.68 indicates that in patients with both factors present, 68% of the PSD risk can be attributed to the interaction between the two factors. This finding has significant clinical implications. It suggests that for patients with preoperative anxiety, postoperative analgesia is not only about relieving pain but also a crucial step in preventing sleep disorders. Conversely, even if the postoperative pain level is moderate, if the patient had significant anxiety before the surgery, the risk of developing PSD would be significantly increased. While the significant additive interaction between anxiety and postoperative pain has clear clinical implications for risk stratification, we acknowledge that quantifying the precise magnitude of risk reduction achievable through specific intervention thresholds (e.g., controlling NRS ≤ 3 in patients with SAS ≥ 50) would require prospective interventional data or counterfactual modeling approaches beyond the scope of this retrospective observational study. Our findings establish the synergistic relationship and identify high-risk subgroups, but cannot directly estimate treatment effects or attributable risk reduction under hypothetical intervention scenarios. Future prospective studies incorporating randomized analgesic protocols or predictive risk calculators would be needed to derive the specific quantitative guidance requested.

Naturally, this study presents several limitations. Firstly, the retrospective design precludes full control of confounding factors, and data completeness depends on medical record quality. Prospective cohort studies with standardized perioperative data collection would reduce information bias. Secondly, PSD was assessed at a single time point (one month post-surgery), capturing mid-term status but not the dynamic evolution across acute and subacute phases. Serial assessments at multiple postoperative intervals are needed to clarify temporal patterns. Thirdly, psychological and sleep evaluations were completed within three days before surgery, a period susceptible to acute hospitalization-related stress; consequently, measurements may reflect situational distress rather than stable baseline traits. Pre-admission or repeated preoperative assessments would help distinguish acute stress from chronic psychological characteristics. Fourthly, this single-center study with 204 patients may limit statistical power, particularly in subgroup and interaction analyses. Multi-center validation with larger cohorts is warranted. Fifthly, all psychological and sleep measures relied on self-reported scales, which may be subject to recall and response bias in elderly patients. Future studies should incorporate objective sleep monitoring (e.g., actigraphy) to complement subjective assessments. Finally, several unmeasured confounders—including postoperative medication details (benzodiazepines, opioids), nighttime ward environmental factors, and post-discharge rehabilitation adherence—may contribute to residual confounding. Despite these limitations, our primary findings regarding preoperative psychological factors remained robust after controlling for early postoperative pain and mobilization timing.

## Conclusions

This retrospective study identified preoperative anxiety, preoperative depression, early postoperative pain, and delayed ambulation as independent risk factors for PSD in elderly patients undergoing hip arthroplasty. Preoperative psychological distress not only independently associated with PSD but also exhibited a significant synergistic interaction with postoperative pain, substantially amplifying the risk. The persistent association between anxiety/depression and PSD across different preoperative sleep status subgroups underscores the fundamental role of mood disorders as trait-like vulnerabilities. Compared with existing literature, this study advances current knowledge by confirming preoperative anxiety and depression as independent predictors of PSD beyond pain and delayed ambulation, demonstrating their significant synergistic interaction with postoperative pain, and validating their consistent predictive value regardless of preoperative sleep status. These findings highlight the necessity of integrating routine preoperative psychological screening and targeted interventions into the perioperative care pathway for this population. A multidisciplinary management strategy addressing both psychological well-being and physiological stressors (pain control and early mobilization) is crucial for mitigating PSD and potentially improving overall recovery outcomes.

## Availability of Data and Materials

All experimental data included in this study can be obtained by contacting the corresponding authors if needed.
